# Developing and evaluating an educational web-based tool for health professions education: the Flipped Classroom Navigator

**DOI:** 10.1186/s12909-022-03647-6

**Published:** 2022-08-01

**Authors:** Punithalingam Youhasan, Marcus A. Henning, Yan Chen, Mataroria P. Lyndon

**Affiliations:** 1 Centre for Medical and Health Sciences Education, Faculty of Medical and Health Sciences, The University of Auckland, Auckland, New Zealand; 2 Department of Medical Education & Research, Faculty of Health-Care Sciences, Eastern University, Sri Lanka, Batticaloa, Sri Lanka

**Keywords:** Flipped classroom, Modern Web, Blended Learning, Educational Technology, Health Profession Education, Web-based interventions, Online educational resource

## Abstract

**Background:**

Flipped classroom pedagogy is a blended learning approach applied in undergraduate health professions education. However, teachers and students may require training to effectively engage in flipped classroom pedagogy. Thus, this study aimed to design, develop, and evaluate a web-based tool for fostering flipped classroom pedagogy in undergraduate health professions education.

**Methods:**

This is an educational design-based research with a descriptive evaluation component which was conducted in two steps: (i) design & development and (ii) evaluation of an educational website. An expert panel was formed to evaluate the website by using a website evaluation questionnaire (WEQ). Descriptive statistics were employed to calculate the experts’ agreement level.

**Results:**

An innovative website design was used to provide access to a range of digital devices. The development process occurred concurrently in two steps: (i) website development and (ii) learning content development. The educational website was branded as the Flipped Classroom Navigator (FCN). Based on WEQ scores, the FCN obtained a good level of agreement (≥ 80%) for its’ ease of use, hyperlinks, structure, relevance, comprehension, completeness, and layout.

**Conclusions:**

The FCN is an effective method for providing training to promote flipped classroom pedagogy in health professions education. The FCN achieved good evaluation scores and comments from experts. However, it is also necessary to obtain acceptance from the end-users, which could be the focus of future research. Nonetheless, the expert panel pinpointed areas for further development before introducing the FCN to end-users.

## Background

Educational practice continues to evolve through technology advancement and blended learning [1]. Blended learning includes face-to-face (F2F) and online teaching–learning components [2]. One of the innovative blended learning approaches is the flipped classroom, where teachers use technology and shift the traditional classroom experience to learners’ own space thus transforming the F2F classrooms into active and applicable educational environments [3, 4]. Flipped classroom pedagogy (FCP) is a relatively new concept and involves using several educational technologies, such as screen and video recorders, a learning management system, online quizzes, and social media platforms [5]. There is growing interest among teachers and students in undergraduate health professional education (HPE) in introductory training in FCP [6, 7]. Thus, it is vital to develop attractive, flexible, and asynchronous training for clinical teachers and students who may be experiencing time challenges when balancing academic and clinical responsibilities [8].

Web-based training (WBT) is popular and recognised as an efficient way to deliver asynchronous training in undergraduate HPE [9, 10]. Such training provides multiple advantages including unrestricted hours of access; freedom from complicated hardware and software; multimedia presentations; asynchronous communication; cost-effectiveness; diverse learning experiences and the opportunity to update content quickly and easily [11, 12].

However, designing and developing an educational website is considerably different from the ordinary website development process [8, 13]. Educational websites are created to activate effective learning rather than being a platform for storing information [14]. Choosing appropriate learning content and a website interface design are crucial for developing an effective educational website [15]. Specifically, three design factors should be considered when developing a high-quality educational website: (i) an *education-oriented aspect* including effective use of multimedia learning principles and appropriate incorporation of learning material with underlying pedagogical principles; (ii) a *user-oriented aspect* consisting of website usability, navigation and accessibility; and (iii) a *multimedia-oriented aspect* incorporating the usage of appealing fonts, typography, texture, colour, graphics, audio and videos [16–19]. Furthermore, Cook and Dupras (2004) proposed practical steps for developing an effective educational website for HPE [13].

Although website development incorporates several design principles, educational websites are predominantly developed from the perspective of the developing team rather than from the learner perspective [20, 21]. Therefore, it is crucial the educational websites are evaluated by learning content experts to consider the educational and learning value of such websites. This study aimed to describe the development and evaluation of an educational website used to train teachers and students in FCP in the undergraduate health professional education setting.

## Methods

### Study design

This was an educational design-based research project with a descriptive evaluation component. This study was conducted in two steps. The first step was designing and developing an educational website for fostering flipped classrooms in an undergraduate health professional education. The second step was focused on evaluating the educational website from the perspective of a panel of content experts.

### Step-1: design and development of an education website

Since the website is created to provide training to health professional educators and students for utilising FCP, we named the educational website “Flipped Classroom Navigator (FCN)”. The core development of the FCN is guided by Cook and Dupras’s [13] practical framework for designing effective educational websites. Cook and Dupras [13] outlined three major essential steps for developing educational websites that employ adult learning principles, namely preparation, development, and implementation and maintenance. The preparation step involves needs analysis, identifying learning outcomes, assessing the opportunity and challenges for the intervention. The development step focuses on creating learning materials and a website skeleton/design. This is conducted by evaluating the nature of the learning content, and whether it promotes active learning, creating strategies for boosting web usage, and evaluating the website before reaching the end-user. Implementation and maintenance is concerned with piloting the website and creating a plan to maintain the website [13].

### Step-2: evaluating the educational website

A quantitative approach was used to appraise the FCN website. An expert panel was formed for evaluating the FCN website. Experts in this study consisted of people who are presently involved in Health Professions Education, Flipped Classroom and Educational Technology as teachers or researchers, or curriculum planners at universities or teaching hospitals. There were ten invitations sent to experts from five different countries. Five experts agreed to participate as an expert panel for evaluating the FCN. The expert panel includes an associate dean for teaching–learning (New Zealand), two senior lecturers (Australia and Sri Lanka), a lecturer in eLearning and web support (New Zealand) and a professional teaching fellow with expertise in flipped classroom pedagogy (New Zealand).

### Data collection and analysis

Several educational website evaluation methods and tools have been proposed to test websites’ usability, the trustworthiness of content, navigation, and layout [22, 23]. The expert panel critically evaluated the FCN website by using the Website Evaluation Questionnaire (WEQ) [23]. The validity and reliability of the WEQ were tested in several contexts [24]. It is a promising instrument (χ2 = 945.7; df = 669; *p* =  < 0.001; CFI = 0.97; NFI = 0.94; RMR = 0.06) that was used to evaluate the FCN [23]. The WEQ consists of 23 items which use a five-point Likert scale with responses ranging from 1 (Most Negative) to 5 (Most Positive). Using the Qualtrics online data collection software (https://tinyurl.com/yfyanost), the WEQ was sent to experts with a URL of the FCN (http://www.flippedclassroom.lk/). Descriptive statistics were employed to analyse experts’ feedback. Responses of 4 (positive) and 5 (most positive) were indicative of high levels of satisfaction. Experts’ agreement rating was calculated for each item by summing the number of agreeable responses divided by the number of experts. An agreement level of 0.75 (75%) or above was considered as good and a value above 0.9 (90%) was indicated as excellent [25, 26].

## Results

The development process includes two activities: website development and learning content development.

### Designing the FCN

This step began with a needs assessment of the FCP in reference to an undergraduate health profession educational context. A baseline survey was conducted to identify the users’ needs and available resources for utilizing FCN [6, 7]. Based on this, intended learning outcomes were developed for the FCN educational website. Then, a core development team was formed, including a web designer, a learning content creator (a health profession educationist), and co-authors (two senior lecturers, and an Associate Professor in medical and clinical education). Regarding content delivery, the website aimed to be compatible for the broadest range of digital devices (including desktop, laptop, smart phone and tablets) and web browsers (including Chrome, Firefox, Internet Explorer and Safari). WordPress software [27] was employed for designing the FCN. The WordPress was used to automatically reformat the shape of the FCN to be compatible with any screen size.

### Developing the FCN

#### Website development

**Fig. 1:**
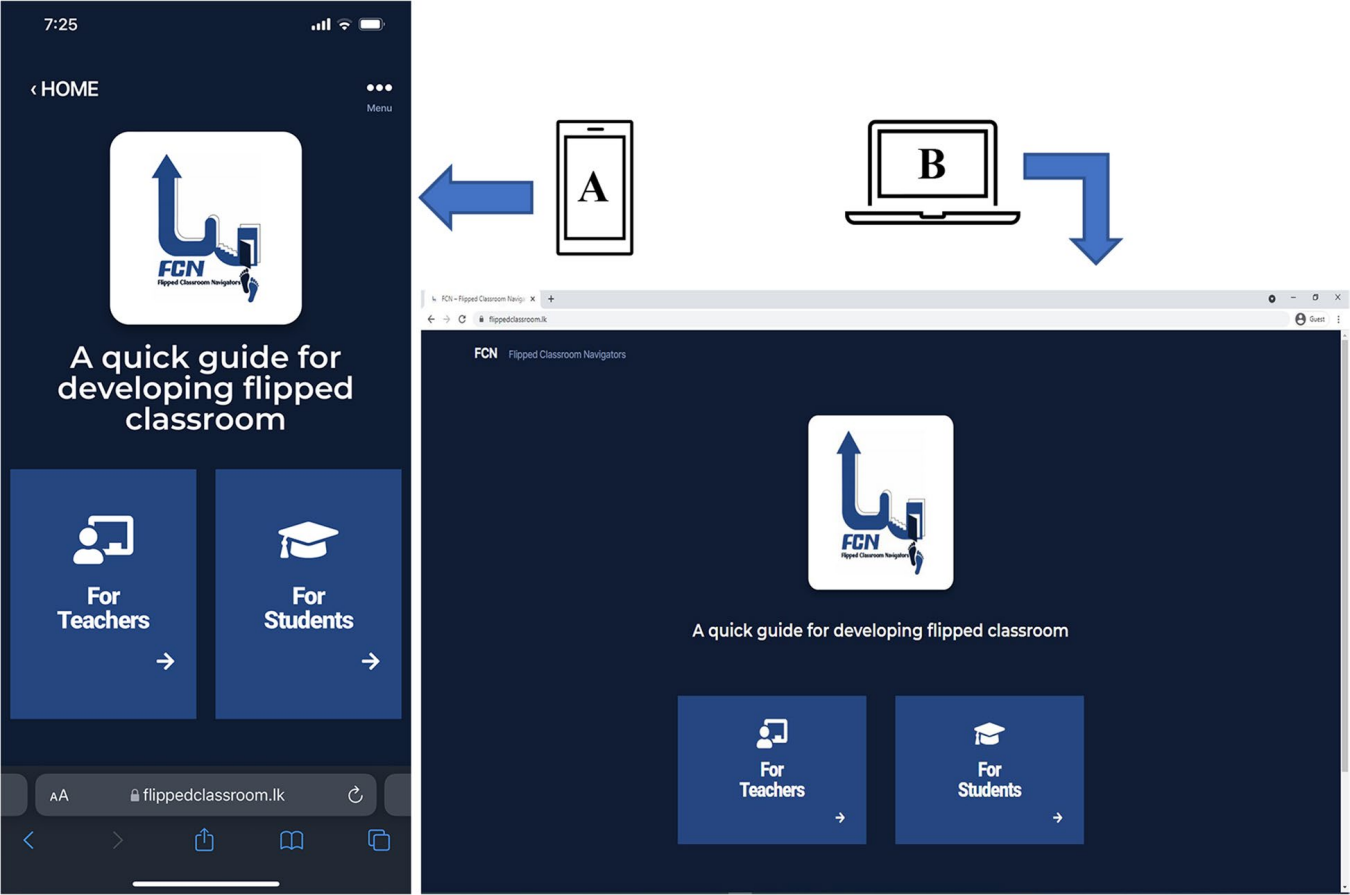
Visual of the FCN home page shown on (**A**) mobile phone and (**B**) laptop

The core development team decided that website design and development go hand in hand with learning content to achieve the intended learning outcomes of the FCN, and the website design needed to be accessible and user-friendly. The FCN web designer developed the user interface and visual layout, which allowed the user to interact with the FCN. A home page was created that could be used by both teachers and students. The home page helps to direct the route that teachers and students could follow to access two different course content pages (See Fig. 1).

**Fig. 2:**
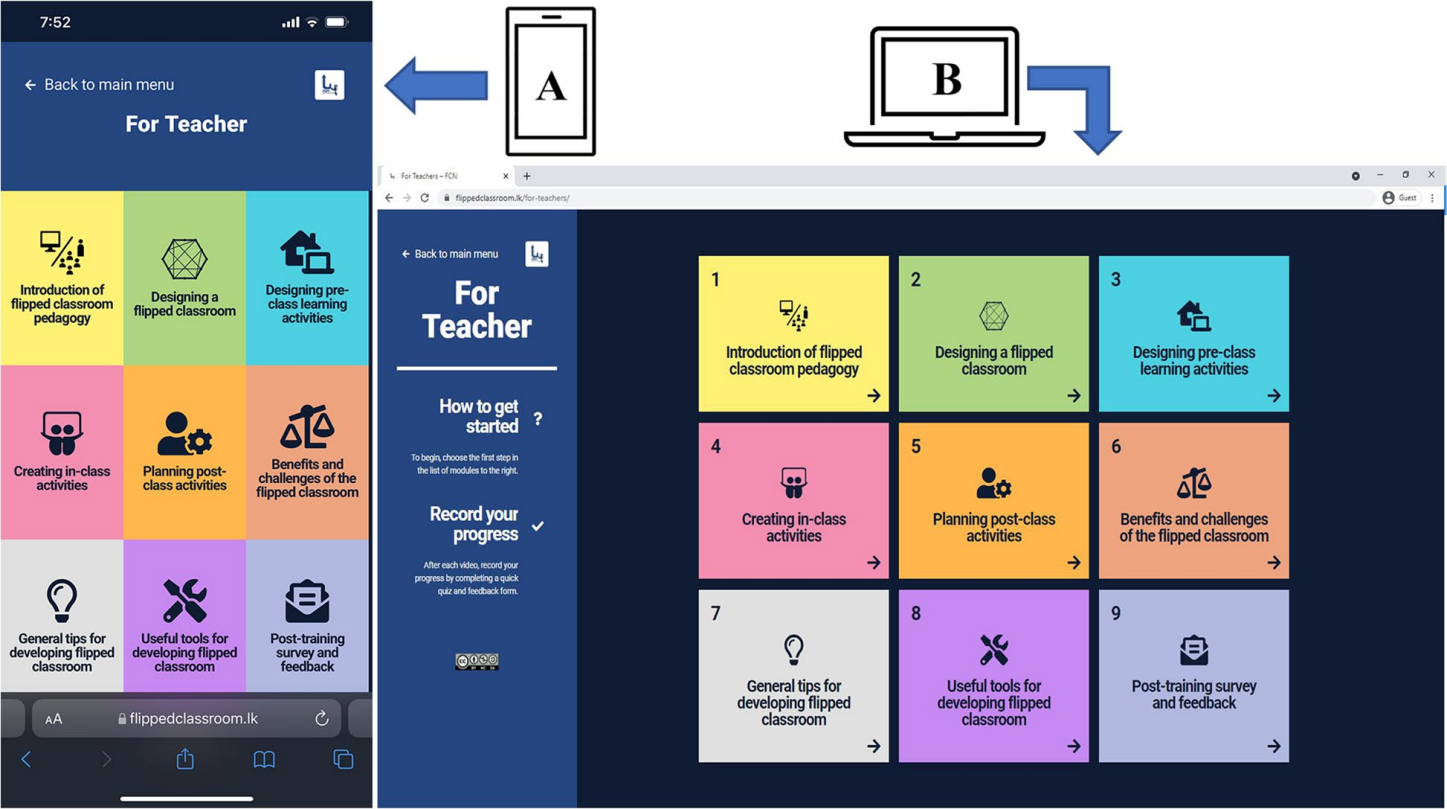
Course content page of the FCN (**A**) mobile phone view and (**B**) laptop view

The course content pages are the most crucial component of the FCN website (See Fig. 2). An innovative tile-based design was used to create coloured rectangles for each topic. Clicking anywhere on the coloured rectangle directs users to the designated topic page (Home Page -> Course Content Page -> Topic page).

**Fig. 3:**
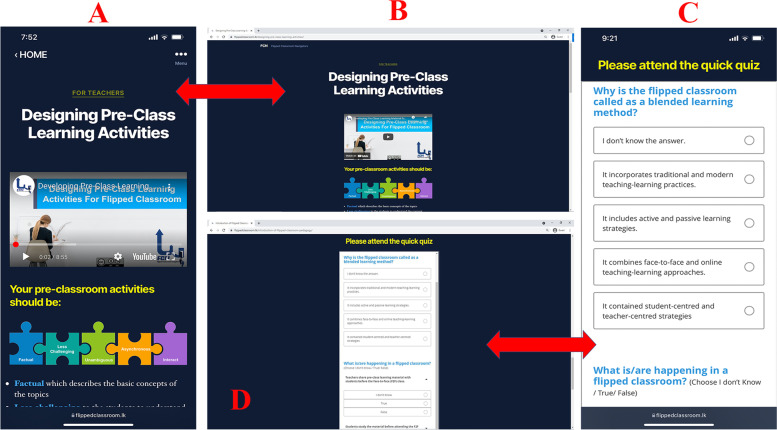
Sample topic page and quizzes of the flipped classroom navigator (**A** & **C**) mobile phone view and (**B** & **D**) laptop view

A common website template was used to create each topic. Each topic page starts with a title, followed by an explanatory video and text or graphic description. A formative assessment (quiz) via Qualtrics was included in each topic for assessing the users’ knowledge gain (See Fig. 3). Users could navigate between FCN pages in three different ways: (i) “home” button on the top left of all pages directs users to the home page; (ii) using “Return to Content” key on the top right of the topic page allows users to coming back to the course content page; and (iii) using arrow keys on the bottom of the topic page allows users to go to the next and previous pages (See Fig. 3).

#### Learning contents development

**Fig. 4:**
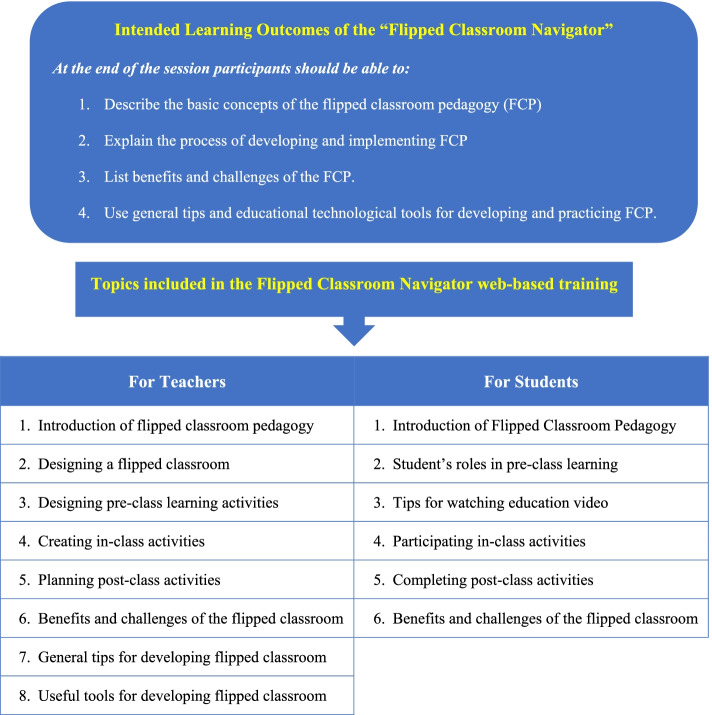
Intended learning outcomes and topics included in the Flipped Classroom Navigator

The learning materials of the FCN were developed in accordance with the intended learning outcomes (ILOs) of the training (see Fig. 4). Constructive alignment informed the development of the learning materials. The core development team thoroughly checked the instructional delivery of the online course (learning content), and quizzes were aligned with ILOs [28, 29]. Eight topics for teachers and six topics for students were created in the online training programme (see Fig. 4).

The course materials were produced in three different forms: word-based illustration (including text explanation, tables, and figures), videos and quizzes. The course materials were developed in three phases: pre-production, production and post-production [30]. In the pre-production phase, the learning content creator (Researcher-1) reviewed relevant literature and drafted scripts for videos and text illustrations. The drafted scripts were cross-checked and reviewed by co-authors for content validity and language suitability.

The production phase started after finalising all scripts. Videos were created using animations, cartoons, and screencasts. Doodly and POWTOON were used to create animations and cartoons [31, 32]. Audacity software was employed in the recording and editing of audio [33]. The video animations and audios were merged and edited in Adobe Premiere Pro to create high-quality (2160p, 4 K) videos [34]. Final videos were uploaded on YouTube (Sample video available for access at: https://youtu.be/TzYhxbXlVjo). The videos were affixed within the FCN by embedding the unique YouTube link. Text illustrations were typed in WordPress. Figures were created in Adobe Photoshop and uploaded into relevant WordPress pages. Quizzes were created in Qualtrics and embedded within WordPress.

In the post-production phase, the drafted WordPress website was internally reviewed by the core development team. In this regard, the core development team met to discuss the quality of learning materials and usability of the website. Several updates and revisions were made in the internal evaluation phase. Finally, the WordPress site was merged with the actual website domain (https://flippedclassroom.lk/). Afterwards, the finalized FCN was shared with the experts’ group for external evaluation.

### Evaluating the FCN

Table-1 provides experts’ responses to the FCN website’s evaluation statements and their agreement rate in percentage. Overall, FCN obtained a good level of agreement from the panel of experts for its features of ease of use, hyperlinks, structure, relevance, comprehension, completeness, layout. Specifically, FCN attained 100% agreement from reviewers in relation to: ‘easy to use’, ‘clear structure’, ‘consistency of sufficient information’.

**Table 1:** Experts’ evaluation of FCN education website

Factor	Statements	Reviewer’s Agreement					Agreement Rate (In %)
		**R1**	**R2**	**R3**	**R4**	**R5**	
**Ease of use**	I find this website easy to use	4	5	5	5	4	**100%**
	*I had difficulty using this website*^*a*^	4	4	5	5	5	**100%**
	I consider this website user friendly	4	5	5	5	3	**80%**
**Hyperlinks**	The homepage clearly directs me towards the information I need	5	5	5	5	3	**80%**
	The homepage immediately points me to the information I need	4	4	4	5	3	**80%**
	*It is unclear which hyperlink will lead to the information I am looking for*^*a*^	5	4	4	5	3	**80%**
	Under the hyperlinks, I found the information I expected to find there	4	4	4	5	3	**80%**
**Structure**	I know where to find the information I need on this website	3	4	5	5	4	**80%**
	*I was constantly being redirected on this website while I was looking for information*^*a*^	5	5	5	5	3	**80%**
	I find the structure of this website clear	4	5	5	5	4	**100%**
	The convenient set-up of the website helps me find the information I am looking for	3	4	4	5	4	**80%**
**Relevance**	I find the information in this website helpful	4	4	5	5	2	**80%**
	*The information in this website is of little use to me*^*a*^	4	5	5	5	3	**80%**
	This website offers information that I find useful	4	4	4	5	3	**80%**
**Comprehension**	The language used in this website is clear to me	4	4	4	5	2	**80%**
	I find the information in this website easy to understand	4	4	5	5	3	**80%**
	*I find many words in this website difficult to understand*^*a*^	4	5	5	5	3	**80%**
**Completeness**	This website provides me with sufficient information	4	4	4	5	4	**100%**
	*I find the information in this website incomplete*^*a*^	4	4	4	5	2	**80%**
	I find the information in this website precise	4	5	5	4	3	**80%**
**Layout**	*I think this website looks unattractive*^*a*^	4	3	5	5	4	**80%**
	I like the way this website looks	4	3	5	4	4	**80%**
	I find the design of this website appealing	4	3	5	4	4	**80%**

^a^ Negative items were recorded in reverse order

Experts also responded to open-ended questions about how to improve each factor. First, the search function needed to be included within the FCN. Second, the latest evidence should be used to support FCP claims mentioned in the FCN. Third, it is helpful to include a glossary page for novice teachers. Fourth, regarding the background colour of the FCN, some users disliked the dark colour of the FCN’s background. Lastly, an expert suggested incorporating transcripts of the FCN’s videos.

## Discussion

The study aimed to design, develop, and evaluate an educational website for promoting and educating the usage of flipped classroom practices in the undergraduate HPE context. Overall, the FCN production was directed by educational website development principles. Expert’s evaluation indicates that the FCN is an effective tool for providing introductory training to promote flipped classroom pedagogy in undergraduate HPE.

It is necessary to follow systematic approaches in developing an educational website for establishing educational principles to achieve meaningful learning outcomes [9, 13]. Adult learning theory principles were incorporated into the learning materials development process for the FCN [8]. For instance, the FCN’s learning materials encouraged self-directed learning and inspired the learner to apply their prior experience. The design of the FCN’s content page was created to provide clear direction and the strongest motivation to participants before they commit to learning it. Moreover, adult learners were inspired by task-oriented education. Therefore, all learning materials were complemented by quick quizzes with instant feedback. In addition, the topics included in the FCN’s educational videos and website were designed as questions forms to trigger the participants’ reflection. For example, “What is happening in a flipped classroom?” and “How the flipped classroom differs from a traditional classroom?”. Participants’ readiness to learn in the FCN was assessed before starting the development process, where they exhibited their willingness to participate in FCP training [6, 7].

The FCN website’s design and development process was directed by Cook and Dupras’s [13] practical guide for developing educational websites. Moreover, the FCN education website design and development process can be explained according to three perspectives. Firstly, from the communication perspective, the FCN was created to train HPE teachers and students by conveying accessible information. Therefore, our core development team decided to choose an accessible website design for transferring our learning materials. This idea was supported by a study indicating that the webpage’s complexity affects communication effectiveness [35]. Secondly, from the aesthetic perspective, the FCN was designed to attract intended users. For example, the contents page was shaped in the form of a colourful grid layout. Further, learning materials were formed as innovative videos and striking graphic illustrations. The rationale being that this promotes timely user interactivity [36]. More specifically it has been reported that users’ initial aesthetic impressions occur within one second of browsing a website [37]. Thirdly, from the human–computer or machine interaction perspective, the FCN can be effectively accessed by several smart devices, including laptops, smartphones, and tablets. Specifically, the webpage template will be automatically transformed according to interactive device screen sizes and their resolution capability. Moreover, the videos and quizzes incorporated in the FCN were designed as interactive tools with the users. The human–computer or machine interaction plays a crucial role in the efficiency and effectiveness of an educational website [38, 39].

The expert panel’s responses to the WEQ indicated a good level of agreement for the FCN for its’ intended use. Specifically, experts agreed on the FCN’s structure, relevance, easy usage, hyperlink accessibility, comprehension, completeness, and layout. In the field of HPE, there are several tools for assessing the quality of educational websites [13, 40]. However, a dearth of evidence has investigated the practice of evaluating education websites before their inaugural launch [41]. Interestingly, the FCN received good to excellent reviews from the content experts before it was made live to the end-users.

Considering the implications of the FCN, it is proposed as a valuable resource for providing flexible and asynchronous learning among the HPE teachers and students who are busy in the academic and clinical environment and can be used as a complement for face-to-face introductory training for implementing FCP.

Educational technological advancements have resulted in several innovative ways to enhance the teaching–learning process, for example, through using educational software. In addition, the inclusion of smart phones as an essential learning gadget, offers further opportunity for incorporating technology into teaching–learning practice through using software and apps. However, developing software is a relatively complex task. In addition, developers usually pay a subscription charge to upload the educational software into various digital platforms for its distribution (e.g., App Store, Google Play and Microsoft Store). To avoid these challenges, a web-based model of delivery was chosen to develop and distribute FCN due to reduced complexity and low cost, easy updating features for educational website developers and free or cost-effectiveness for end-users. Another strength of the FCN is its straightforward and user-friendly structure which provides unambiguous navigation to users, which was supported by the experts’ evaluation that FCN’s was clearly structured and accessible (culminating in a 100% agreement).

Considering future improvements, firstly, the FCN development team can focus on addressing the feedback from the expert panel, namely attending to background colour, evidence-based practice for supporting FCN claims, and incorporating the suggested enhancements. These include technical changes to the search function, glossary page and transcripts of the FCN’s videos. Secondly, actual end-users (teachers and students from undergraduate HPE) need to use the FCN and, therefore, be able to provide feedback regarding their experience when using the FCN. Thirdly, few learners prefer to read the learning materials in a text book format [21]. Therefore, the FCN development team can explore the possibility to automatically convert the website format as a reader-friendly text, book, or use audiobook instructions. Lastly, it is also possible to create a space for sharing users flipped classroom experiences in the FCN and enabling the users to share the web-blog writings on their website or social media.

## Conclusion

The FCN is a web-based educational tool designed and developed to provide asynchronous training to teachers and students in undergraduate HPE. The design and development of the FCN was guided by the educational principles of effective learning. The FCN educational website obtained a good level of agreement from an international expert panel. More specifically, FCN received 80 to 100% acceptance from experts for it being easy to use, having a clear structure and being consistency of sufficient information. We believe that FCN would add value to delivering online induction training for practising FCN. However, the effectiveness of the FCN will be confirmed after being rolled out to the end-users.

## Data Availability

All relevant data are within the manuscript.

## Abbreviations

F2F: Face-to-Face
FCP: Flipped Classroom Pedagogy
HPE: Health Professional Education
WBT: Web-based training
FCN: Flipped Classroom Navigator
WEQ: Website Evaluation Questionnaire
